# Completeness of the disease recording systems for dairy cows in Denmark, Finland, Norway and Sweden with special reference to clinical mastitis

**DOI:** 10.1186/1746-6148-8-131

**Published:** 2012-08-06

**Authors:** Cecilia Wolff, Mari Espetvedt, Ann-Kristina Lind, Simo Rintakoski, Agneta Egenvall, Ann Lindberg, Ulf Emanuelson

**Affiliations:** 1 Department of Clinical Sciences, Swedish University of Agricultural Sciences, P.O. Box 7054, SE-750 07, Uppsala, Sweden; 2 Department of Production Animal Clinical Science, Norwegian School of Veterinary Science, P.O. Box 8146 Dep, NO-0033, Oslo, Norway; 3 Department of Large Animal Sciences, Faculty of Health and Medical Sciences, University of Copenhagen, Grønnegårdsvej 8, 1870, Frederiksberg, Denmark; 4 Department of Veterinary Biosciences, University of Helsinki, P.O. Box 66, FI-00014, Helsinki, Finland; 5 National Veterinary Institute, SE-751 89, Uppsala, Sweden

**Keywords:** Bovine mastitis, Disease recording, Completeness, Nordic, Database, Validation

## Abstract

**Background:**

In the Nordic countries Denmark, Finland, Norway and Sweden, the majority of dairy herds are covered by disease recording systems, in general based on veterinary registration of diagnoses and treatments. Disease data are submitted to the national cattle databases where they are combined with, e.g., production data at cow level, and used for breeding programmes, advisory work and herd health management. Previous studies have raised questions about the quality of the disease data. The main aim of this study was to examine the country-specific completeness of the disease data, regarding clinical mastitis (CM) diagnosis, in each of the national cattle databases. A second aim was to estimate country-specific CM incidence rates (IRs).

**Results:**

Over 4 months in 2008, farmers in the four Nordic countries recorded clinical diseases in their dairy cows. Their registrations were matched to registrations in the central cattle databases. The country-specific completeness of disease registrations was calculated as the proportion of farmer-recorded cases that could be found in the central database. The completeness (95% confidence interval) for veterinary-supervised cases of CM was 0.94 (0.92, 0.97), 0.56 (0.48, 0.64), 0.82 (0.75, 0.90) and 0.78 (0.70, 0.85) in Denmark, Finland, Norway and Sweden, respectively. The completeness of registration of all CM cases, which includes all cases noted by farmers, regardless of whether the cows were seen or treated by a veterinarian or not, was 0.90 (0.87, 0.93), 0.51 (0.43, 0.59), 0.75 (0.67, 0.83) and 0.67 (0.60, 0.75), respectively, in the same countries. The IRs, estimated by Poisson regression in cases per 100 cow-years, based on the farmers’ recordings, were 46.9 (41.7, 52.7), 38.6 (34.2, 43.5), 31.3 (27.2, 35.9) and 26.2 (23.2, 26.9), respectively, which was between 20% (DK) and 100% (FI) higher than the IRs based on recordings in the central cattle databases.

**Conclusions:**

The completeness for veterinary-supervised cases of CM was considerably less than 100% in all four Nordic countries and differed between countries. Hence, the number of CM cases in dairy cows is underestimated. This has an impact on all areas where the disease data are used.

## Background

In intensive dairy production, high-quality disease data are a valuable resource. The Nordic countries Denmark (DK), Finland (FI), Norway (NO) and Sweden (SE) have established national milk recording schemes where data from disease recording are combined with data from milk recording and data on reproductive events in a central database. The central databases are managed by the farmer-owned dairy co-operatives the Danish Cattle Federation [1], the Agricultural Data Processing Centre (FI) [2], TINE SA (NO) [3] and the Swedish Dairy Association [4]. The information is used by herd managers, in advisory work and breeding programmes, and for research. The disease information in the databases is mainly based on records made by veterinarians. In all four Nordic countries included, the legislation concerning veterinary care restricts the availability of prescription drugs, such as antibiotics, for both parenteral and local use, in dairy cattle. Therefore, in general, when a farmer detects a cow with clinical mastitis (CM), she or he must consult a veterinarian who makes a treatment decision after clinical examination.

**Figure 1:**
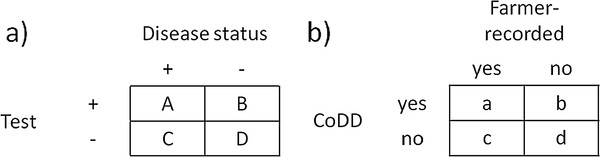
**Sensitivity and completeness formulas.a**. Sensitivity of a diagnostic test is calculated as follows: A/(A + C). Cell D represents healthy animals correctly classified as test-negative. **b**. Completeness of the disease information in each of four national cattle databases (CoDDs) was calculated as a/(a + c). The Table covers cases of disease (not animals). Cell d represents cases of clinical mastitis not recorded by participating farmers or in the CoDD.

Completeness of a disease database is defined in terms of the proportion of true disease events that are also recorded in the database and is consequently similar to the sensitivity of a diagnostic test [5]. Referring to Figure 1a, completeness is therefore calculated as A/(A + C). Theoretically, if the cattle disease recording systems function well, the probability that all veterinary-diagnosed or -treated disease events are captured should be high. However, by design none of these disease recording systems will include 100% of the clinical disease events as some will not warrant a veterinary consultation, which is more or less a prerequisite for recording. 

In a previous study, where disease registrations from the Nordic countries were analysed in a comparable manner, the incidence rates (IRs) for CM were markedly different [6]. Further, in a similar study using raw data from all four countries, discrepancies in between-country incidence risks of veterinary-treated CM were seen [7]. The Swedish dairy disease recording system has been subject to validation in earlier studies. Mörk et al. [8] found a significant difference between the IRs based on reports by farmers and IR based on registrations in the central database. A comparison of copies of records left on-farm by the veterinarian (i.e. concerning events that had been veterinarian-attended) with registrations of veterinary examinations in the central database showed a completeness for diagnostic events of 75% [9]. Furthermore, a Danish comparison between written registrations made by veterinarians and registrations in the Danish database revealed that 18–21% of diagnoses were missing in the database at the individual cow level [10]. This leads to the hypothesis that the completeness of disease registrations in the central databases, also for CM, may differ between the four Nordic countries included, which may in part explain the differences in the incidences of CM.

The primary aim of this study was to estimate the completeness of disease registrations, with the diagnosis CM in focus, in the four Nordic cattle databases. A second aim was to estimate the CM IRs, as detected and recorded by farmers, by country.

## Methods

### Disease recording in the Nordic countries

According to the legislation in the Nordic countries, veterinarians should record when a cow is treated with any drug with a withdrawal period, e.g. any antibiotics. In NO and SE, veterinarians are required to write a record regardless of whether the cow diagnosed with a disease was treated or not. In SE, the veterinarian needs to submit the record to the Board of Agriculture ( http://www.sjv.se). If the herd participates in milk recording the information is subsequently transferred to the Swedish Dairy Association’s cattle database. In DK and NO, recording of all diseases, i.e. the submission of the disease record to a central database, is mandatory for herds in milk recording. In FI about 90% of the herds in milk recording also participate in disease recording, to have access to herd health and breeding advisory services. In DK, the farmer or the veterinarian submits the records to the central database. In FI, the veterinarian records the data on the cow’s health card and an artificial insemination (AI) technician or the farmer submits the records to the central database. In NO, the farmer, herd management advisor or veterinarian submits the records. In all four countries, farmers can also record diseases themselves with or without involvement of a veterinarian. For a detailed description of the disease recording systems, see, e.g., Olsson et al. [11], Bartlett et al. [12], Gröhn et al. [13], Østerås et al. [14] and Mörk et al. [9].

There are two exceptions to the rule that clinically diseased cows may not be treated with prescription drugs without prior clinical examination by a veterinarian. The first is the “common practice” for milder cases of CM in FI. Finnish dairy producers may take a milk sample and send it for bacteriological analysis, and consult the veterinarian when the results are known. Depending on the veterinarian’s judgement, treatment may be prescribed without the veterinarian visiting the farm. The farmer should record any treatment on the cow’s health card; the information is then submitted to the central database as described above. The second exception is the Danish system for veterinary care in dairy cattle that was introduced in 2006 and by 2008 was used by approximately 8% of Danish dairy farmers [15]. Farmers may, after signing a contract with their veterinarian, initiate treatment themselves for certain disorders and only with a predetermined drug of choice. Instead of visiting each diseased cow, the veterinarian makes frequent scheduled visits to examine all cows at specific and predetermined stages of lactation and to follow up previously treated cows. In this system, dairy cows can be regarded as veterinary-treated since the diagnostic criteria and follow-up of treatment are under veterinary supervision. Diagnoses and treatments made by the farmer are recorded and submitted to the database by the farmer. We hereafter refer to this system as the “Danish New Herd Health (DNHH)”.

### Study herds and data collection

The data collection for CM was conducted as a part of a larger study where data for four major production-related complexes where studied and where data collection was conducted in parallel for all four complexes (mastitis, lameness, and metabolic and reproductive disturbances). The number (%) of dairy herds in milk recording in DK, NO and SE was approximately 4,000 (90%), 11,800 (97%) and 5,000 (76%) herds, respectively, in 2008. In FI, the health surveillance system kept records of approximately 8,700 (70%) herds in the same year, 2008. The target population for this study were cows belonging to herds participating in these national schemes with a herd size of at least 15 cows. Four random samples were selected from these populations. Country-specific sample size calculations were done, with the disease event as the unit of interest. Calculations were based on an expected completeness of 80%, country-specific average herd sizes, the national registered disease incidences of previous years and a maximum width of the confidence intervals of 0.1. To account for the unknown clustering of registered disease events within herds, the sample size was doubled. The target sample size was 150–200 herds per country, which was considered conservative. Taking country-specific expected response rates and practical circumstances into account, 1,000, 900, 800 and 400 herds in DK, FI, NO and SE, respectively, were invited to participate. The owners of these herds were sent a letter explaining the purpose of the study and giving a description of what was expected of participating herds. In FI, NO and SE, participants were offered lottery tickets with prizes ranging from a gift voucher for travel (FI) and weekend travel to an agricultural show (NO) to free embryos (SE). In DK, no incentives were offered to participating farmers.

Data collection was done during two study periods in 2008, from 15 February to 15 April and from 15 September to 15 November. In DK, the first study period started and ended 2 weeks later for practical reasons. The study periods were chosen to avoid recording during the most labour intensive months for the farmers, but to still capture possible seasonal variation. Participating farmers were provided with written information, recording sheets, and prepaid envelopes for returning the recording sheets. Recording sheets could also be sent by fax or e-mail. In FI, an online recording option was available. All farmers were given a reminder by telephone or text message after 1 month and at the end of each study period. Where no disease events were detected, farmers reported “no events” either by returning a recording sheet with only the herd identification (ID), or by sending a text message, fax or email.

Before the second period all farmers were contacted by mail or phone and provided with new recording sheets, instructions and envelopes. Farmers who did not want to continue participating after the first study period, or who had failed to return their recording sheets or report absence of disease events were excluded. In DK and NO, a few farmers participated only during the second study period.

A number of parameters for the participating herds and target populations of herds were investigated to assess how representative the study herds were. The selected parameters represented milk production, udder health, reproductive performance and indicators of metabolic status. Due to limited data access, official country statistics [16] and only mean values without measures of spread were used for DK instead of target population data.

### The farmer-recorded data

The diagnosis and clinical signs of mastitis, i.e. ‘redness, soreness or swelling of the udder’ and ‘visible changes of the milk’, as well as systemic signs, e.g. “fever’ and ‘poor appetite’, were listed on the recording sheets with a tick-box next to each item. Farmers had to fill in the herd ID, cow ID, date of detection of symptoms, dates of veterinary visits, who diagnosed the cow (farmer or veterinarian), and any treatments the cow was given or absence of treatment. All CM events were recorded at the cow-level (quarter-specific data were not recorded). A copy of the recording sheet, in English or any of the four Nordic languages concerned, is available from the main author upon request.

Instructions were printed on the back of each recording sheet. Only cows were included in the study, i.e. no heifers or young stock. If a cow recovered, but had a relapse, a new recording sheet was filled out. In general, the same recording sheet was used for all revisits by a veterinarian for the same disease event, and all therapy for that event, etc. Only events of clinical disease were recorded, i.e. where the cow was not well according to the farmers’ judgement. Events of subclinical mastitis, e.g. high somatic cell counts (SCCs) at test milking or at California Mastitis Test (CMT) testing, were not recorded as ‘CM’. No additional cow-side test was used, unless such tests were part of the normal routines in the herd.

Data from the submitted recording sheets were manually entered into an Access database (DK and SE) or electronically scanned (FI and NO). In FI, web-based recording was also available. All records were manually checked for data entry errors. Records were removed if the date of detection was outside the study period (n = 25), if no dates were noted (n = 36), or if the herd ID or cow ID was missing or unknown (n = 14). All data management and analysis was done using the statistical package SAS 9.2 (SAS Institute Inc., Cary, NC, USA).

A disease event was defined as “CM” if one of the following was indicated by the farmer on the recording sheet: “mastitis”, “changed appearance of the milk“or “swelling, redness or soreness of the udder” following the International Dairy Federation’s definition [17]. A CM event was defined as “veterinary-visited” if one of the following was indicated: “veterinary-examined”, “diagnosed by a veterinarian”, “treated by a veterinarian” or if a date for a veterinary visit was stated. For herds participating in the DNHH system (n = 10), CM events were also defined as “veterinary-visited” if the farmer had recorded “treatment with antibiotics or non-steroid anti-inflammatory drugs by the farmer”, because such treatments were under veterinary supervision. For the same reason, the definition for “veterinary-visited” CM events in the Finnish data was extended to antibiotics given by the farmer, to include cows that were treated after analysis of a milk sample, and after a telephone consultation and prescription by the veterinarian.

All CM events that occurred within 8 days of the first event were considered one CM case [18]. The date used for defining a case was the first visit date even though each recording sheet could have several dates including date of detection and several visits dates. If no visit date was given on a recording sheet, the date of detection was used. If any of the events within a case were defined as “veterinary-visited”, the entire case was defined as “veterinary-visited”. For the case date, the date of the first event was used.

### Data from the national cattle databases

Data from the four national cattle databases were retrieved in May 2009 and rearranged into one common disease database (CoDD). The retrieved information included cow-level information on entries and removals, reproductive events, milk recording and disease records. Disease records included events during the time from 7 days before to 7 days after the two study periods. Diagnostic codes differed between the countries. For CM, seven, three, two and 14 codes were used in DK, FI, NO and SE, respectively, which represented varying degrees of clinical signs or other characteristics of CM (Table 1). To make a comparison between countries possible, all diagnostic codes for CM were re-coded to “clinical mastitis”. Disease events had a diagnostic date according to the veterinarian’s (or farmer’s) recording. Also in the CoDD, all events with the diagnostic code CM within 8 days of the first event were viewed as one mastitis case, with the case date being the date of the first event.

**Table 1:** The country-specific diagnostic codes for clinical mastitis in 2008

Denmark	11 Mastitis, 12 Mastitis during dry period, 14 Mastitis following teat lesion, 15 Acute mastitis, 72 Summer mastitis, 94 Toxic mastitis, 179 Mastitis with paresis
Finland	301 Acute clinical mastitis, 303 Chronic mastitis, 610 Owners notes: Mastitis during lactation
Norway	303 Clinical mastitis, severe or moderate, 304 Clinical mastitis, mild
Sweden	2101 and 2102 Acute mastitis, 2103 Mastitis, 2104 and 9765 Reoccurring mastitis, 2116 Chronic mastitis, 2117 and 9779 Exacerbating clinical mastitis, 2147 Teat lesion with mastitis, 9764 Acute clinical mastitis, 9766 Mastitis with gangrene, 9767 Mastitis with sepsis, 9769 Chronic clinical mastitis, 9789 Teat lesion with clinical mastitis

### Completeness

**Figure 2:**
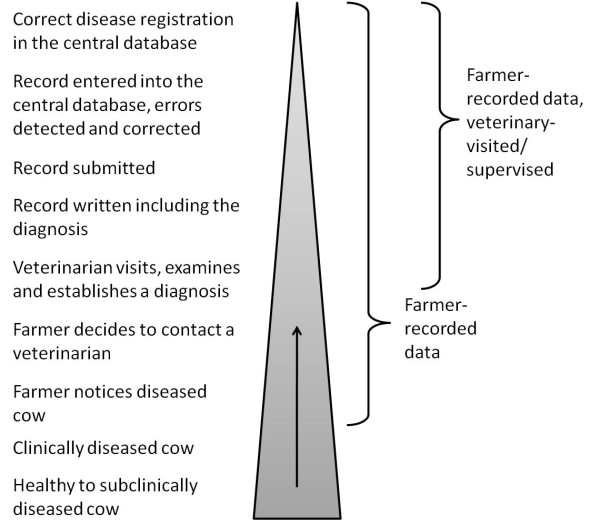
Illustration of the data flow from the diseased dairy cow to the national cattle databases in the four Nordic countries included in the study.

The CM cases in the CoDD were matched to CM cases in the farmer-recorded data. A match was defined as the same country, herd ID and cow ID with either the exact same case date (n = 1597), or allowing a discrepancy of 7 days between the case dates (n = 223). Larger date discrepancies were evaluated. Completeness was calculated by country as the proportion (p) of farmer-recorded cases (n) that could be matched to a CoDD case, i.e., referring to Figure 1b, as a/(a + c). The completeness was calculated separately for all cases in the farmer-recorded data and for cases defined as “veterinary-visited” (Figure 2). Double-sided confidence intervals for the completeness at a significance level of 95% (z = 1.96) were calculated as follows:

(1) $$\text{p}+/-\text{z}\surd \left(\text{p}\left(1-\text{p}\right)/\text{n}\right)$$

To account for the unknown clustering of cases within herds, the confidence intervals were increased post hoc [19] by factor 2.

Cases of CM that were present only in the CoDD and not in the farmer-recorded data were considered not properly recorded by farmers in the study, rather than being considered errors in the CoDD. In an alternative calculation of completeness these cases were reclassified as if they had also been recorded by the farmers, i.e. completeness = (a + b)/(a + c + b) (Figure 1b). We hereafter refer to the farmer-recorded data (FRD) with the excess CoDD CM cases added as “adjusted FRD”.

If the number of CM cases in the FRD divided by the number of CM cases in the CoDD was >0.9 the herd was classified as a herd where the farmer was a good reporter of findings. Consequently, for herds with many excess CM cases in the CoDD, farmers were classified as poor reporters. The completeness calculations were repeated, this time including only good reporters.

For the Finnish FRD, an additional completeness calculation was done where antibiotics administered by the farmer were not included as a criterion for a CM case to be defined as “veterinary-visited”, i.e. using the same definition for “veterinary-visited” as in NO, SE and DK (excluding herds in the DNHH).

### Incidence rate calculation

The time-at-risk for CM was defined as follows: in the CoDD, cows were given one record per lactation and disease case, meaning that cows with no mastitis events had one record per lactation. When a cow left or re-entered the herd during the study period the observation period for that cow ended or started on that date. If a cow calved during the study period, the new lactation, and consequently a new observation period, started on the calving date. The previous observation period ended the day before the calving date, i.e. the dry period was included in the time-at-risk. Heifers were included from the date of first calving. Hence, if a cow both entered and left the herd on the same day she contributed with 1 day at risk during which she could have a registered a disease event.

Country- and database-specific IRs for CM were estimated by Poisson regression (PROC GENMOD, SAS 9.2) with a deviance scale parameter to account for overdispersion in the data. The different dependent variables were the number of CM cases per herd in the CoDD, or veterinary-visited cases or all cases in the farmer-recorded data. Days at risk per herd was the offset variable. The days at risk were the number of cow-days per herd during the two study periods reduced by 8 days per CM case in the FRD or CoDD. Negative binomial models were also evaluated. The changes in point estimates and confidence intervals compared to the Poisson models were minor and did not affect the differences in IRs between countries or between datasets within countries.

## Results

The number of study herds (% of invited herds) was 105 (11%), 167 (19%), 179 (22%) and 129 (32%), of which 79, 132, 159 and 118 participated during both study periods in DK, FI, NO, and SE, respectively. The parameters representing production and health in the study herds were judged as being comparable to those of the target populations (Table 2). Descriptive statistics of the farmer-recorded data are presented in Table 3. In all four countries, the most common second diagnosis (>50% of all second diagnoses) in a cow with CM was teat lesions (Table 3). The number of such events was 27, 31, 33 and 46 in DK, FI, NO, and SE, respectively. The percentage of cows with CM and no other disease in the herd on a particular date was 80%, 90%, 94% and 85% in DK, FI, NO and SE, respectively. The frequencies of disease and mastitis events in the CoDD are presented in Table 3.

**Table 2:** Characteristics of the study herds and target populations (Finland, Norway and Sweden) or all dairy herds (Denmark)

	**Denmark**		**Finland**		**Norway**		**Sweden**	
	**Study herds**^**a**^	**All dairy herds**^**a**^	**Study herds**	**Target population**	**Study herds**	**Target population**	**Study herds**	**Target population**
	**Mean**	**Mean**	**Median (p25; p75)**^**b**^	**Median (p25; p75)**^**b**^	**Median (p25; p75)**^**b**^	**Median (p25; p75)**^**b**^	**Median (p25; p75)**^**b**^	**Median (p25; p75)**^**b**^
Average herd size (cow-years)	109	126	26.4 (20.8; 37.8)	25.4 (19.7; 35.5)	21.4 (18.1; 27.9)	21.2 (17.6; 27.6)	49.2 (33.9; 74.5)	45.4 (30.8; 69.2)
Age at first calving (months)	–	–	25 (25; 27)	25 (25; 26)	25.8 (24.7; 27.3)	25.6 (24.5; 27.1)	27.6 (26.4; 29.3)	27.8 (26.3; 30.0)
Calving to first insemination (days)	–	–	92 (83; 104)	93 (83; 106)	76 (69; 84)	78 (70; 86)	89 (80; 99)	91 (81; 105)
Average yearly milk yield per cow (kg)	8,914	8,922	9,122 (8,372; 9,796)	8,738 (7,991; 9,443)	7,233 (6,434; 7,805)	7,008 (6,366; 7,622)	9,446 (8,671; 10,047)	9,313 (8,516; 9,999)
SCC status geometric mean^d^ (1,000 cells)	241	245	154 (108; 202)	156 (114; 209)	127 (101; 155)	130 (104; 159)	218 (184; 281)	226 (177; 284)
Fat percentage (%)	4.41	4.26	4.16 (3.94; 4.38)	4.22 (4.00; 4.43)	4.15 (3.96; 4.39)	4.41 (3.92; 4.43)	4.19 (4.04; 4.34)	4.18 (4.04; 4.33)
Protein percentage (%)	3.47	3.41	3.44 (3.38; 3.51)	3.45 (3.38; 3.51)	3.39 (3.34; 3.46)	3.39 (3.32; 3.46)	3.41 (3.34; 3.50)	3.14 (3.33; 3.49)

The target populations were herds in milk recording with an average herd size of at least 15 cows. The figures are only for within-country comparison since the presented parameters are calculated differently for each country.

^a^ The access to target population data from Denmark was limited. Consequently, officially published country statistics and only mean values are presented without measures of spread.

^b^ 25^th^ and 75^th^ percentiles.

^c^ We had no access to this figure for all dairy herds in Denmark.

^d^ The Swedish mean is arithmetic. In Denmark the mean is of the bulk milk SCC, in the other three countries the mean is calculated from individual cows SCC.

SCC = somatic cell count.

**Table 3:** Descriptive statistics of clinical mastitis (CM) registration and cow information in farmer-recorded data (FRD) and retrieved from the national cattle common disease databases (CoDDs) in four Nordic countries in 2008

	**Denmark**		**Finland**		**Norway**		**Sweden**	
	**FRD**	**CoDD**	**FRD**	**CoDD**	**FRD**	**CoDD**	**FRD**	**CoDD**
Number of cows		13,548		6,287		6,387		8,896
Cow-years		3,118		1,566		1,552		2,316
Number of herds with CM (% of study herds)	98 (93)	99 (94)	136 (81)	105 (63)	131 (73)	121 (68)	106 (81)	102 (71)
Median number of CM cases per study herd (p10, p90)^a^	6 (1; 17)	9 (2; 27)	2 (0; 8)	1 (0; 5)	2 (0; 5)	1 (0; 5)	3 (0; 9)	1 (0; 8)
Number of cows with CM	873	1,031	492	270	357	326	478	371
Number of CM cases (% veterinary-visited)^b^	938 (85)	1,218	536 (89)	301	379 (87)	359	498 (83)	404
Number of farmer-recorded (FR) CM events (% with a second diagnosis^c^ on the recording sheet)	974 (4.8)		545 (11.2)		391 (12.8)		511 (11.7)	

^a^ 10^th^ and 90^th^ percentiles.

^b^ All cases in the CoDD are seen by a veterinarian and/or are veterinary-treated.

^c^ Other diagnosis than CM.

Overall, the completeness of registration of CM cases was highest in the Danish data and lowest in the Finnish data (Table 4). The completeness (95% confidence interval) for veterinary-visited cases ranged from 0.90 (0.86, 0.94) in DK to 0.50 (0.41, 0.59) in FI. The point-estimates of completeness increased slightly in all countries when the adjusted FRD was used to 0.94, 0.56, 0.82, 0.78 in DK, FI, NO and SE, respectively (Table 4). Also the proportion of veterinary-treated cases increased by 8%, 2%, 3% and 4% in DK, FI, NO and SE, respectively when the adjusted FRD was used. Increasing the allowed discrepancy in the number of days between a farmer-recorded CM case and a matched CoDD case from 7 to 30 days increased the proportion of matches by 2%, 4%, 3% and 3% in DK, FI, NO and SE, respectively. When only herds with good reporting were included in the FRD, the completeness decreased (Table 4).

**Table 4:** Country-specific completeness (95% confidence interval) of registration of farmer-recorded (FRD) clinical mastitis (CM) cases in four national databases, calculated as the proportion of FR CM cases found in a common disease database (CoDD) when allowing a date discrepancy of 7 days

**Dataset**		**Denmark**	**Finland**	**Norway**	**Sweden**
Original FRD	All cases	0.85 (0.80; 0.90)	0.45 (0.32; 0.58)	0.68 (0.57; 0.80)	0.61 (0.50; 0.72)
	Veterinary-visited cases	0.90 (0.86; 0.94)	0.50 (0.41; 0.59)	0.77 (0.68; 0.86)	0.72 (0.63; 0.81)
Adjusted FRD^a^	All cases	0.90 (0.87; 0.93)	0.51 (0.43; 0.59)	0.75 (0.67; 0.83)	0.67 (0.60; 0.75)
	Veterinary- visited cases	0.94 (0.92; 0.97)	0.56 (0.48; 0.64)	0.82 (0.75; 0.90)	0.78 (0.70; 0.85)
Adjusted FRD^b^	All cases	0.80 (0.73; 0.87)	0.46 (0.37; 0.54)	0.67 (0.58; 0.77)	0.60 (0.51; 0.69)
	Veterinary- visited cases	0.90 (0.84; 0.95)	0.51 (0.42; 0.60)	0.76 (0.67; 0.86)	0.72 (0.63; 0.81)

The CoDD consisted of information retrieved from the four Nordic national cattle databases. The study was carried out during two 2-month periods in 2008.

^a^ Excess CoDD cases added to the FRD.

^b^ Only herds with good reporting to the FRD were included. The number of herds with good reporting was 48/105 (46%), 148/167 (89%), 142/179 (79%) and 105/129 (81%) in Denmark, Norway, Finland and Sweden, respectively.

CI = confidence interval. FRD = farmer-recorded data.

In addition, the Finnish CM data were analysed separately, without defining antibiotic treatments by the farmer as “veterinary-visited”. The number of veterinary-visited CM cases was then reduced by 51 to 426. After matching these to CoDD data, the completeness (95% confidence interval) for veterinary-visited cases (allowing a data discrepancy of 7 days) increased from 0.50 (0.41, 0.59) to 0.53 (0.43, 0.62).

The IRs in the adjusted FRD and CoDD differed, based on point estimates and confidence intervals, both within and between the countries (Table 4). In SE, the IR (95% confidence interval) for all cases in the adjusted FRD was 26.2 (23.2, 29.6) compared to 17.5 (14.9, 20.6) in the CoDD. In FI, both the IR for all cases and veterinary-visited cases was higher than the IR in the CoDD (Table 5). The IRs from the adjusted FRD in DK were significantly higher than in NO and SE, and the IRs in FI were higher than in SE, for both veterinary-visited cases and all cases.

**Table 5:** Incidence rates (IRs) estimated by a Poisson model for clinical mastitis (CM) cases as registered in four Nordic countries’ national common disease databases (CoDDs) for cattle, and recorded by farmers, with excess cases in the CoDDs added (adjusted FRD)

**IR (95% CI) – cases per 100 cow-years**	**Denmark**	**Finland**	**Norway**	**Sweden**
CoDD	39.4 (35.4; 43.9)	19.3 (16.4; 22.8)	23.2 (19.8; 27.3)	17.5 (14.9; 20.6)
Adjusted FRD; all cases	46.9 (41.7; 52.7)	38.6 (34.2; 43.5)	31.3 (27.2; 35.9)	26.2 (23.2; 29.6)
Adjusted FRD; veterinary- visited cases	42.1 (37.4; 47.5)	34.7 (30.6; 39.4)	28.1 (24.3; 32.6)	22.6 (19.7; 25.8)

The study was carried out during two 2-month periods in 2008.

CI = confidence interval. FRD = farmer-recorded data.

## Discussion

The results support our hypothesis that the completeness of the disease recording systems, both for veterinary-visited cases and for the total number of cases of CM, differs between the four Nordic countries included in this study. The different completeness figures and IRs retrieved in this study indicate that DK’s disease recording system captured the highest proportion of CM cases. Also, DK had the highest IR of CM both in the FRD and in the CoDD. The FRD IR for FI was higher than that for both NO and SE, which in official statistics based on the CoDD is hidden by the poor completeness. Although the completeness was better in NO than in SE, SE had the lowest IRs.

Given the design of the disease recording systems in the Nordic countries the completeness of the data for veterinary-visited cases should, theoretically, be 100%, which was not the case in any country. This leads to an underestimation of the incidence of veterinary-visited CM in all four Nordic cattle databases, which has implications for all areas where this information is used.

What can be regarded as an acceptable level of completeness for a disease database depends on the purpose of the recording system, and how the data are used. For example, in research using secondary data (i.e. data collected for purposes other than the research in question), such as the disease data evaluated here, it is essential to know the proportion of disease in the population under investigation captured by the recording system and database. Moreover, for any study aiming at a between-country comparison of disease frequency, knowledge of differences in data quality is necessary to obtain valid results.

The number of studies validating a disease database for production animals is very limited. In a Swedish study, including all ages and diagnoses of dairy cattle, 71% of farmer-reported (FR) events could be found in data from the cattle database [8]. The Norwegian disease recording system reportedly underestimates calf morbidity by, on average, 40% [20]. In Denmark, about one-fifth of reporting of diseases by veterinarians on individual cows could not be found in the cattle database [10]. The findings in the present study support these previous studies.

### Completeness

The reason for imperfect completeness of registration of veterinary-visited cases of CM is that one or several of the steps in the disease recording system (i.e. recording, submission, transfer or entry into the database) do not work properly (Figure 2). In FI, the common practice for mastitis cases where systemic signs are absent is that the farmer first takes a milk sample; once the bacteriological results are available, he or she consults the veterinarian. If the cow is treated with antibiotics from a phone prescription, the farmer is instructed to record this on the cow’s health card. The low completeness in Finland indicates that this, generally, is not fully complied with. Consequently, the disease recording system fails to capture approximately every second CM case in Finnish dairy herds. This is in agreement with Saloniemi [21] who estimated the proportion of non-recorded CM cases in Finland treated with phone prescription antibiotics to be 65%. When the Finnish CM cases that were obviously treated with over-the-phone prescription antibiotics were classified as “non-veterinary-visited” the completeness increased slightly. Unfortunately, the FRD was in many cases not detailed enough to distinguish between veterinary and farmer-administered treatment with phone prescription antibiotics. A significant within-country difference in completeness for veterinary-visited cases could also be expected at regional or even local level since neighbouring herds may use different veterinarians and advisory services whose recording and submission rate may differ [9,22]. This was, however, not assessed in the present study. The FRD also did not allow any analysis of the characteristics of the FR cases that could not be found in the CoDD, e.g. with milder or more severe clinical signs.

One step down in the data flow from the diseased cow to the database (Figure 2) is the farmer’s threshold for detection, which will also affect the completeness of the information in the database. A farmer with a low threshold for detection, but not necessarily for treatment, will notice many CM cases but not have them treated by a veterinarian, and thereby decrease the total completeness. The completeness for all CM cases, regardless of whether they are veterinary-visited or not, depends both on the completeness of registration of the veterinary-visited cases and on the overall proportion of CM cases that are veterinary-visited, i.e. the coverage of the disease recording system. The lower the coverage, the lower the completeness for all cases, since the proportion of cases expected to be captured by the disease recording system decreases. The coverage is highly influenced by the farmers’ threshold for consulting a veterinarian. The cases where the farmer chooses to consult a veterinarian, i.e. cases that should be in CoDD, are likely to be more severe compared to cases where the veterinarian is not contacted or the cow not treated with prescription drugs; the coverage would be higher for severe CM cases compared to milder cases. For cases in the CoDD there is no tool to assess the severity of a CM case other then what diagnostic code the veterinarian used when there are several to choose from (Table 1). The FRD recordings were, in general, not detailed enough to assess the degree of severity for veterinary-supervised cases compared to non-veterinary-supervised cases. It has been shown that dairy producers may have different thresholds for contacting a veterinarian depending on their attitudes to mastitis [23,24], but the decision is also influenced by the herd situation and cow factors for that specific case, in particular when the cow presents only milder symptoms [25]. In the present study, the high completeness both for veterinary-visited cases and for all cases in DK and the low completeness figures in FI could not be explained by a high versus low coverage.

In this study, disease occurrence as noted by the farmers during their normal routines was used as the standard against which the CoDD data were compared. Mastitis is a common disorder in dairy cows and any dairy producer should be familiar with its clinical manifestation. However, the individual herdsman’s threshold for detection depends on several factors, such as the diagnostic tools used, e.g. cow-side tests or detection via the milking system. Some routine tests will detect subclinical disease as well. We chose not to include any cow-side tests, such as the CMT, as a criterion for the farmers to use in this study, since not all farmers use this in their everyday work and some have not used it at all. Also, we wanted the farmers’ recording of disease events to be as close to their normal level of detection as possible, without influencing their threshold for detection or their threshold for contacting their veterinarian since this could have influenced the completeness.

In the presence of a gold standard for true disease status, completeness of a database is calculated similarly to the sensitivity of a diagnostic test, or A/(A + C) in Figure 1a. To fully assess the accuracy of a database, the correctness = A/(A + B) in Figure 1a, equivalent to the positive predictive value of a diseased animal, should be checked. In the present study the unit of interest was the disease case, i.e. completeness was calculated for CM cases (Figure 1b), not diseased animals (Figure 1a). Correctness could not be calculated in this study because the farmers obviously did not record 100% of all veterinary-visited (or -supervised) CM cases in the FRD; had they done so, the adjusted FRD would have been identical to the original FRD and cell b in Figure 1b empty. This was illustrated when we included only herds belonging to good reporters of findings; the difference between completeness for the original FRD and the adjusted FRD was minor (Table 4). In other words, the number of CM cases that were not recorded in either the FRD or in the CoDD (cell d in Figure 1b) was unknown.

In DK, the number of farmer-recorded CM cases (938) was much lower than the number of CM cases in the CoDD (1,218) (Table 3), indicating that the Danish farmers did not report all CM events they detected, in the FRD. Any CM cases recorded in the CoDD are most likely to have been observed by the farmer at some point, even if they were not recorded in the FRD, and the adjustment of the FRD was done with the assumption that the disease information in the CoDD is correct although not complete. Hence, the addition of excess CoDD cases to the FRD should not lead to any false FR cases. However, the adjusted FRD led to an overestimation of the completeness for all CM cases, particularly in DK because of the Danish farmers’ lower-than-expected reporting to the FRD. It was possible for a herd with many veterinary-visited cases not recorded in the FRD to also have many non-veterinary-visited cases that were not recorded in the FRD (cell d in Figure 1b), but the extent of these events could not be estimated in our study.

### Incidence rates

The country-specific differences between incidences based on the adjusted FRD with veterinary visits versus the CoDD reflect the completeness for cases seen by a veterinarian. For example, in NO, these figures were 28.1 and 23.2, respectively, and the corresponding completeness was estimated to be 0.82. The overall IR, including all cases, in the adjusted FRD should be close to the true observable occurrence of CM. Also, the IR of cases seen by a veterinarian based on the adjusted FRD should capture all cases receiving veterinarian attention except those that were not recorded in either the FRD or the CoDD. Accordingly, the overall IR in the adjusted FRD will be an underestimation of the true IR since the adjustment to the FRD only influences cases seen by a veterinarian (cell b in Figure 1b) and any cases not seen by a vet and not recorded in the FRD are unknown (cell d in Figure 1b).

The incidence of CM has previously been estimated to be 36–48 cases per 100 cow-years in DK [12], with 17% lactational risk in FI [26], 21.3 cases per 100 cow-years in NO [14], and 22.6 veterinary-treated cases per 100 cow-years in SE [8]. Non- Nordic authors have reported incidence rates of 23.0 cases per 100 cow-years in Canada [27], 44.1 and 20.1 cases per 100 cow-years in France [28,29], 47 cases per 100 cow-years in England and Wales [30], 25.2-27.8 cases per 100 cow-years in the Netherlands [31]. The IRs that were estimated in the present study based on the FRD suggest that the incidence of CM in the Nordic countries is higher than what has been found in previous studies but within the range of what has been estimated in non-Nordic countries. However, comparing incidence between and within countries as estimated in research studies is problematic. Incidence rates may not be comparable due to different inclusion criteria or definitions of what makes a CM case. A direct comparison of official disease incidence between countries is not accurate either since the process that generates such figures is unique to each country. Nevertheless, the IRs from the CoDD roughly agree with the previous Nordic studies. In the present study the CoDD data were extracted as raw data that were re-coded, edited and presented identically in all four countries, which has allowed us to use the IRs calculated in this study for a between-country comparison.

There might be seasonal variation in CM occurrence which may impact on estimates of IR based on only two study periods. However, there were no such trends for the IR estimates detected from FRD and CoDD (data not shown); some estimates were slightly higher during the autumn period but some were lower. Seasonal variation in the IRs in this study might be a true reflection of the occurrence of CM or effects of variation in recording compliance by farmers (both FRD datasets), variation in the completeness of recordings in the CoDD, or because proportionally fewer cows were visited by a veterinarian (veterinary-visited FRD and CoDD). The completeness estimates did not differ between study periods (data not shown).

### Study herds

When the completeness calculation included only herds with good reporting to the FRD, the completeness decreased, both for all cases and for veterinary-visited CM only. This was due to an increase in the number of cases captured only in the FRD (cell c in Figure 1b). In other words, these cases increased the denominator in the completeness formula, which consequently led to a decrease in the calculated completeness. Since the estimate of completeness for the good reporters is likely to be closer to the “true” completeness, the overall completeness figures for the FRD presented in this study are likely to be overestimated.

A large number of herds were invited to the study in DK, FI and NO to achieve the desired sample size. For practical reasons, it was not possible to first invite a smaller sample of herds and then contact non-responders individually, as was done in SE. For this type of study, where highly motivated farmers are crucial, we were willing to accept the proportionally low response rate this caused. Nevertheless, this may have introduced selection bias, e.g. with regard to herd size, udder health, production level, or farmers who were dissatisfied with the quality of disease data or who had a particular interest in disease recording or research studies. However, our study herds did not deviate to a large extent from target herds with respect to characteristics related to size and performance (Table 2). A fair number of herd owners who agreed to participate did not do so, or participated in only one of the two study periods (with the majority of these participating only in the first study period). We did not try to persuade these farmers to continue participating but tried to include farmers who were willing to do a good job with recording.

## Conclusions

The completeness for farmer-recorded CM cases, either veterinary-visited cases only or including all cases, ranged between 0.51 and 0.94. Denmark had the highest completeness and FI the lowest. Our study shows that the IR for CM is underestimated in all four included countries when it is based on disease data from the cattle databases. This underestimation affects genetic evaluations, advisory work, statistics and research. The IRs for CM also differed between farmer recordings and the cattle databases. Overall, the highest FR IR was found in DK and the lowest in SE. The largest percentage point difference between FR and CoDD IR was found in FI, reflecting poor completeness of the database in that country.

## Competing interests

The authors declare that they have no competing interests.

## Authors’ contributions

CW, ME, AKL and SR participated in the study design and were responsible for data collection in their respective countries. CW further performed the statistical analysis and drafted the manuscript. AL, AE and UE participated in the study design and statistical analyses and revised the manuscript. SR, ME and AKL revised the manuscript. All authors have read and approved the final manuscript.

## References

[B1] Registrering og Ydelsekontrol (RYK) http://www.landbrugsinfo.dk/Kvaeg/RYK/Sider/Startside.aspx

[B2] Maatalouden Laskentakeskus http://www.mloy.fi/MLWeb/FI/default.html

[B3] Helsetjenesten for storfehttp://storfehelse.tine.no/

[B4] Kokontrollen basen för beslut http://www.svenskmjolk.se/Mjolkgarden/Service--radgivning/Ko-kontrollen/

[B5] Hogan WR Wagner MM Accuracy of data in computer-based patient records J Am Med Inf Assoc 1997 4 342 355 10.1136/jamia.1997.0040342 PMC61252 9292840

[B6] Plym-Forshell K Osterås Ø Aagaard K Kulkas L Saran A, Soback S Disease recording and cell count data in 1993 in Sweden, Norway, Denmark and Finland. Progress in the control of mastitis Proceedings of the 3rd International Mastitis Seminar, 28 May – 1 June 1995; Tel Aviv, Israel 1995 50 54

[B7] Valde JP Lawson LG Lindberg A Agger JF Saloniemi H Østerås O Cumulative risk of bovine mastitis treatments in Denmark, Finland, Norway and Sweden Acta Vet Scand 2004 45 201 210 10.1186/1751-0147-45-201 15663080 PMC1820994

[B8] Mörk M Lindberg A Alenius S Vagsholm I Egenvall A Comparison between dairy cow disease incidence in data registered by farmers and in data from a disease-recording system based on veterinary reporting Prev Vet Med 2009 88 298 307 10.1016/j.prevetmed.2008.12.005 19178966 PMC7114122

[B9] Mörk MJ Wolff C Lindberg A Vagsholm I Egenvall A Validation of a national disease recording system for dairy cattle against veterinary practice records Prev Vet Med 2010 93 183 192 10.1016/j.prevetmed.2009.09.016 19819035

[B10] Bennedsgaard TW Reduced Use of Veterinary Drugs in Organic Dairy Herds - Potentials and Consequences 2003 Royal Veterinary and Agricultural University of Copenhagen, Department of animal husbandry

[B11] Olsson SO Baekbo P Hansson SO Rautala H Østerås O Disease recording systems and herd health schemes for production diseases Acta Vet Scand Suppl 2001 94 51 60 11875853 10.1186/1751-0147-42-S1-S51 PMC8041038

[B12] Bartlett PC Agger JF Houe H Lawson LG Incidence of clinical mastitis in Danish dairy cattle and screening for non-reporting in a passively collected national surveillance system Prev Vet Med 2001 48 73 83 10.1016/S0167-5877(00)00192-6 11154781

[B13] Gröhn Y Saloniemi H Syväjärvi J An epidemiological and genetic study on registered diseases in Finnish Ayrshire cattle. I. The data, disease occurrence and culling Acta Vet Scand 1986 27 182 195 3799396 10.1186/BF03548163 PMC8189415

[B14] Østerås O Solbu H Refsdal AO Roalkvam T Filseth O Minsaas A Results and evaluation of thirty years of health recordings in the Norwegian dairy cattle population J Dairy Sci 2007 90 4483 4497 10.3168/jds.2007-0030 17699070

[B15] Anonymous Evaluering af ordningen om Ny Sundhedsrådgivning i Kvægbesætninger. (Evaluation of the regulation on cattle New Herd Health Advisory schemes) [in Danish] http://www.ft.dk/samling/20072/almdel/flf/bilag/377/580729.pdf

[B16] Danish Cattle Federation Figures on Danish Cattle 2009 http://www.landbrugsinfo.dk/kvaeg/filer/kvaegbruget_tal2009eng.pdf

[B17] International Dairy Federation Suggested interpretation of mastitis terminology Bull IDF 1999 338 3 26

[B18] International Dairy Federation Recommendations for presentation of mastitis-related data Bull IDF 1997 321 6 24

[B19] McDermott JJ Schukken YH Shoukri MM Study design and analytic methods for data collected from clusters of animals Prev Vet Med 1994 18 175 191 10.1016/0167-5877(94)90074-4

[B20] Gulliksen SM Lie KI Østerås O Calf health monitoring in Norwegian dairy herds J Dairy Sci 2009 92 1660 1669 10.3168/jds.2008-1518 19307648

[B21] Saloniemi H Udder diseases in dairy cows – field observations on incidence, somatic and environmental factors, and control J Sci Agric Soc Finland 1980 52 85 184

[B22] Sviland S Waage S Clinical bovine mastitis in Norway Prev Vet Med 2002 54 65 78 10.1016/S0167-5877(02)00014-4 12062520

[B23] Nyman AK Ekman T Emanuelson U Gustafsson AH Holtenius K Waller KP Sandgren CH Risk factors associated with the incidence of veterinary-treated clinical mastitis in Swedish dairy herds with a high milk yield and a low prevalence of subclinical mastitis Prev Vet Med 2007 78 142 160 10.1016/j.prevetmed.2006.10.002 17092590

[B24] Jansen J Van den Borne BHP Renes RJ Van Schaik G Lam TJGM Leeuwis C Explaining mastitis incidence in Dutch dairy farming: the influence of farmers’ attitudes and behaviour Prev Vet Med 2009 92 210 223 10.1016/j.prevetmed.2009.08.015 19800700

[B25] Vaarst M Paarup-Laursen B Houe H Fossing C Andersen HJ Farmers’ choice of medical treatment of mastitis in Danish dairy herds based on qualitative research interviews J Dairy Sci 2002 85 992 1001 10.3168/jds.S0022-0302(02)74159-3 12018446

[B26] Rajala PJ Gröhn YT Disease occurrence and risk factor analysis in Finnish Ayrshire cows Acta Vet Scand 1998 39 1 13 9592941 10.1186/BF03547802 PMC8050666

[B27] Olde Riekerink RGM Barkema HW Kelton DF Scholl DT Incidence rate of clinical mastitis on Canadian dairy farms J Dairy Sci 2008 91 1366 1377 10.3168/jds.2007-0757 18349229

[B28] Fourichon C Beaudeau F Bareille H Seegers H Incidence of health disorders in dairy farming systems in western France Livest Prod Sci 2001 68 157 170 10.1016/S0301-6226(00)00249-9

[B29] Barnouin J Bord S Bazin S Chassagne M Dairy management practices associated with incidence rate of clinical mastitis in low somatic cell score herds in France J Dairy Sci 2005 88 3700 3709 10.3168/jds.S0022-0302(05)73056-3 16162545

[B30] Bradley AJ Leach KA Breen JE Green LE Green MJ Survey of the incidence and aetiology of mastitis on dairy farms in England and Wales Vet Rec 2007 160 253 258 10.1136/vr.160.8.253 17322356

[B31] Barkema HW Schukken YH Lam TJGM Beiboer ML Wilmink H Benedictus G Brand A Incidence of Clinical Mastitis in Dairy Herds Grouped in Three Categories by Bulk Milk Somatic Cell Counts J Dairy Sci 1998 81 411 419 10.3168/jds.S0022-0302(98)75591-2 9532494

